# The Stringent Response Promotes Antibiotic Resistance Dissemination by Regulating Integron Integrase Expression in Biofilms

**DOI:** 10.1128/mBio.00868-16

**Published:** 2016-08-16

**Authors:** Emilie Strugeon, Valentin Tilloy, Marie-Cécile Ploy, Sandra Da Re

**Affiliations:** Inserm, UMR1092, Université de Limoges, UMR-S1092, and CHU Limoges, Laboratoire de Bactériologie-Virologie-Hygiène, Limoges, France

## Abstract

Class 1 integrons are genetic systems that enable bacteria to capture and express gene cassettes. These integrons, when isolated in clinical contexts, most often carry antibiotic resistance gene cassettes. They play a major role in the dissemination of antibiotic resistance among Gram-negative bacteria. The key element of integrons is the integrase, which allows gene cassettes to be acquired and shuffled. Planktonic culture experiments have shown that integrase expression is regulated by the bacterial SOS response. In natural settings, however, bacteria generally live in biofilms, which are characterized by strong antibiotic resilience and by increased expression of stress-related genes. Here, we report that under biofilm conditions, the stringent response, which is induced upon starvation, (i) increases basal integrase and SOS regulon gene expression via induction of the SOS response and (ii) exerts biofilm-specific regulation of the integrase via the Lon protease. This indicates that biofilm environments favor integron-mediated acquisition of antibiotic resistance and other adaptive functions encoded by gene cassettes.

## IMPORTANCE

Multidrug-resistant bacteria are becoming a worldwide health problem. Integrons are bacterial genetic platforms that allow the bacteria to capture and express gene cassettes. In clinical settings, integrons play a major role in the dissemination of antibiotic resistance gene cassettes among Gram-negative bacteria. Cassette capture is catalyzed by the integron integrase, whose expression is induced by DNA damage and controlled by the bacterial SOS response in laboratory planktonic cultures. In natural settings, bacteria usually grow in heterogeneous environments known as biofilms, which have very different conditions than planktonic cultures. Integrase regulation has not been investigated in biofilms. Our results showed that in addition to the SOS response, the stringent response (induced upon starvation) is specifically involved in the regulation of class 1 integron integrases in biofilms. This study shows that biofilms are favorable environments for integron-mediated acquisition/exchange of antibiotic resistance genes by bacteria and for the emergence of multidrug-resistant bacteria.

## INTRODUCTION

Antibacterial drugs are one of the most important therapeutic advances in medical history, but bacterial resistance has increased dramatically over the last decade. Multidrug-resistant (MDR) Gram-negative bacteria are spreading worldwide and are becoming a major public health issue. Clinicians are now dealing with infections for which very few effective antibiotics are available. The question, therefore, is how to resist resistance and thereby preserve the effectiveness of existing antibiotics. In addition to preventing antibiotic overuse, we urgently need to better understand how bacteria acquire and disseminate determinants of antibiotic resistance (1, 2).

Along with transposons and plasmids, integrons are important genetic elements involved in the dissemination of antibiotic resistance among Gram-negative bacteria (3, 4). The integron’s functional platform is composed of a gene encoding an integron integrase, *intI*, a specific recombination site, *attI*, and a promoter, Pc, which controls the expression of promoterless genes embedded within gene cassettes (5). The integrase catalyzes gene cassette insertion and excision through site-specific RecA-independent recombination (6). Hundreds of classes of integrons have been described on the basis of the amino acid sequence of the IntI protein; they are found in all ecosystems (human, animal, and environment), providing to bacteria multiple adaptive functions (7–9). In clinical settings, five classes of integrons have been described, which mainly contain antibiotic resistance gene cassettes (7). The class 1 integrons are those most commonly encountered in human commensals and pathogens. Integrons containing antibiotic resistance gene cassettes are usually located on mobile genetic elements (plasmids or transposons) (10). More than 130 gene cassettes have been described, conferring resistance to almost all antibiotic classes (11).

Integrase expression is regulated by the bacterial SOS response (12). This coordinated response to DNA damage requires a repressor, LexA, and a sensor/activator, RecA (13, 14). During normal bacterial growth, LexA is bound at attachment sites (SOS boxes) in the promoter region of genes of the SOS regulon, which comprises at least 43 unlinked genes in *Escherichia coli* (15, 16). In response to DNA damage that leads to single-stranded DNA (ssDNA) formation, ssDNA-RecA nucleoprotein filaments induce LexA autoproteolysis (17), thereby releasing promoters and enabling gene expression. Among the stresses that can induce the SOS response, several antibiotics, as well as horizontal gene transfer events like transformation and conjugation, have been shown to enhance integrase expression and activity in planktonic cultures of *E. coli* and *Vibrio cholerae* (12, 18–20). In addition to SOS response regulation, the nucleoid-associated proteins FIS and H-NS were recently suggested to repress the expression of IntI1 (21). The *V. cholerae* integron integrase (IntIA, formerly called IntI4) was also shown to be controlled by cyclic AMP (cAMP) receptor protein (CRP)-dependent regulation (19).

All of these regulatory mechanisms have been extensively studied in planktonic culture, whereas in natural settings, bacteria mostly live in biofilms. A biofilm is a community of microbes associated with a biotic or abiotic surface, typically encased in an autoproduced extracellular matrix (22). Biofilms are characterized by high levels of antibiotic resistance/tolerance compared to those of their planktonic counterparts and represent a major health threat when they develop during chronic infections or on medical devices (23). The antibiotic resilience of bacterial biofilms results from a variety of mechanisms (24, 25). Recalcitrance (or tolerance) is mainly due to the presence of an isogenic subpopulation of nondividing, antibiotic-tolerant bacteria called persisters (26, 27). The SOS and stringent responses are the two main pathways leading to the generation of persister bacteria (24). Recently, Bernier et al. showed that starvation and SOS response induction in aging biofilms mediated bacterial tolerance to fluoroquinolones (28). Biofilms are highly heterogeneous environments with local gradients of nutrients, pH, oxygen tension, etc., creating microniches of distinct bacterial subpopulations that experience and adapt to various stresses (29, 30). Another characteristic explaining the survival of biofilm bacteria during antibiotic exposure is that biofilms facilitate the transfer of mobile genetic elements and, therefore, the spread of antibiotic resistance between bacteria (31–34). It has been shown that various environments where bacteria live in complex biofilms contain large numbers of integrons displaying a huge variety of gene cassettes (35–37).

We therefore studied the influence of the biofilm lifestyle on class 1 integron integrase expression by comparing the expression levels o*f intI1* and the recombination activities of the IntI1 integrase in planktonic and biofilm culture. We found that the stringent response acts at two levels in biofilms: it induces the SOS response, thereby increasing the basal expression level of SOS-regulated genes, and also it exerts biofilm-specific positive regulation of *intI1* expression through a mechanism involving the Lon protease.

## RESULTS

### The SOS response and integrase expression are induced by the biofilm lifestyle.

In a continuous-culture biofilm model, we examined the expression level of the *intI1* gene and that of *sfiA*, a gene that encodes the cell division inhibitor SulA and is known to be strongly induced by the SOS response. We used *E. coli* MG1656 F′ (a strain with a strong propensity to form biofilms, due to the presence of the F′ factor [38]) and plasmid pPsfiA-*lacZ* or pPintI1-*lacZ*, carrying a *lacZ* transcriptional fusion with, respectively, the promoter of *sfiA* (PsfiA) or *intI1* (PintI1) (Table 1). We first compared the promoter activities by assaying β-galactosidase in MG1656 F′ cells grown for 24 h under planktonic and biofilm conditions. The MG1656 F′/pPsfiA-*lacZ* and MG1656 F′/pPintI1-*lacZ* strains exhibited, respectively, 2.2- and 3.6-fold higher β-galactosidase activity under biofilm conditions than in planktonic culture (Table 2, B/P ratio).

**TABLE 1:** Bacterial strains and plasmids used in this study

Strain or plasmid	Relevant genotype or description	Reference or source
*E. coli* strains		
MG1656	*lacZ* null derivative of *E. coli* MG1655	69
MG1656 Δ*recA*	Deletion of the *recA* gene; constitutive repression of SOS genes	12
MG1656 Δ*sulA* Δ*lexA*	Deletion of the *lexA* and *sulA* genes; constitutive expression of SOS genes	12
NEB 5-alpha F′ Iq	F′ *proA*^+^*B^+^ lacI*^q^ Δ(*lacZ*)M15 *zzf*::Tn*10* (Tet^r^)/*fhuA2*Δ(*argF-lacZ*)U169 *phoA glnV44* φ80Δ(*lacZ*)M15 *gyrA96 recA1 relA1 endA1 thi-1 hsdR17*	New England Biolabs
TG1 Δ*relA*::KmFRT	Deletion of *relA* by replacement of the gene with a KmFRT cassette; Km^r^	28
TG1 Δ*cpxR*::KmFRT	Deletion of *cpxR* by replacement of the gene with a KmFRT cassette; Km^r^	70
TG1 Δ*rpoS*::KmFRT	Deletion of *rpoS* by replacement of the gene with a KmFRT cassette; Km^r^	28
TG1 Δ*luxS*::KmFRT	Deletion of *luxS* by replacement of the gene with a KmFRT cassette; Km^r^	28
TG1 Δ*lon*::KmFRT	Deletion of *lon* by replacement of the gene with a KmFRT cassette; Km^r^	28
MG1656 Δ*cpxR*	Deletion of *cpxR*	This study
MG1656 Δ*rpoS*	Deletion of *rpoS*	This study
MG1656 Δ*luxS*	Deletion of *luxS*	This study
MG1656 Δ*lon*	Deletion of *lon*	This study
MG1656 Δ*relA* Δ*spoT*	Deletion of *relA* and *spoT*	This study
Plasmids		
p6851	Cassette excision reporter pSU38::*aac(6′)-Ib*::*attC_aadA7_*-*cat*(T4)-*attC*_VCR2_; Km^r^ Cm^r^	12
pSU38Δtot*lacZ*	Vector carrying the *lacZ* coding sequence with no translation initiation region or promoter; Km^r^	68
pPsfiA-*lacZ*	*sfiA* promoter cloned into pSU38Δtot*lacZ*: *lacZ* under the control of PsfiA; Km^r^	This study
pPintI1	*attI* site from In40 class 1 integron cloned into pSU38Δtot*lacZ*: *lacZ* under the control of the *intI1* promoter, PintI1; Km^r^	12
pPintI1*	pPintI1 with PintI1 carrying the mutation LexAmut2 in the LexA box; Km^r^	12
pZE1-mcs1	Promoterless derivative of pZE12-mcs1; Amp^r^	12
pZE1-IntI1	*attI* site + *intI1* gene from In40 class I integron (integrase IntI1_R32_H39_ variant with the highest excision activity); Amp^r^	12
pZE1-IntI1*	pZE1-IntI1 carrying the mutation LexAmut2 in the LexA box of PintI1; Amp^r^	12
pCP20	Vector carrying Flp gene specific to FRT sites, thermosensitive; Amp^r^ Cm^r^	66
F′	F′ conjugative plasmid allowing enhanced biofilm formation; Tet^r^	38
pZS*tetR11-mcs1	Plasmid carrying P_N25_-*tetR* between the *bla* gene and the terminator t0 and the synthetic P_LtetO-1_ promoter in front of the multiple-cloning site MCS1, pSC101* origin of replication; Amp^r^	This study
pZS*tetR11-*relA*	Same as pZS*tetR11-mcs1 but with *relA* under the control of the synthetic P_LtetO-1_ promoter; Amp^r^	This study
pZS*tetR11-*lon*	*lon* under the control of the synthetic P_LtetO-1_ promoter; Amp^r^	This study

**TABLE 2:** *intI1* and *sfiA* expression under biofilm conditions versus planktonic culture

Strain^a^	β-Gal activity (Miller units) [mean (±SD)^a^ or B/P ratio] in strain bearing indicated plasmid under indicated condition(s)								
	pPintI1-*lacZ*			pPintI1*-*lacZ*			pPsfiA-*lacZ*		
	P	B	B/P	P	B	B/P	P	B	B/P
MG1656 F′	17.6 (±4.3)	64.0 (±24.3)	3.6#	97.6 (±23.2)	202.9 (±29.3)	2.1#	584.1 (±288.9)	1270.6 (±469.1)	2.2#
MG1656 Δ*lexA* F′	74.1 (±8.2)	109.5 (±17.8)	1.5#	76.9 (±17.6)	135.3 (±14.5)	1.8#	16858.7 (±4,084.4)	15647.9 (±3,517.5)	0.9, NS
MG1656 Δ*recA* F′	6.4 (±0.7)	8.4 (±1.7)	1.3#	ND	ND		1.1 (±0.1)	2.3 (±0.1)	2.1, NS

a The results are from at least 12 replicates. P, planktonic culture; B, biofilm; #, significant difference at a *P* value of <0.001; ND, not determined; NS, not significant.

To determine whether PsfiA and PintI1 induction under biofilm conditions is linked to SOS-dependent regulation, we measured the β-galactosidase activities of both promoters in the MG1656 F′ Δ*recA* (constitutive repression) and Δ*lexA* (constitutive expression) deletion mutant derivatives (Table 1). In biofilm culture, the PsfiA and PintI1 activities were dramatically reduced in strain MG1656Δ*recA* F′ (557- and 7.6-fold, respectively) (Table 2) and increased in strain MG1656Δ*lexA* F′ (12.3- and 1.7-fold, respectively) (Table 2) compared to their activities in the wild-type strain. Thus, basal *sfiA* and *intI* expression (expression level in the absence of exogenous stress) was higher under biofilm conditions than in planktonic culture, nevertheless allowing both promoters to be further activated by the SOS response.

**FIG 1:**
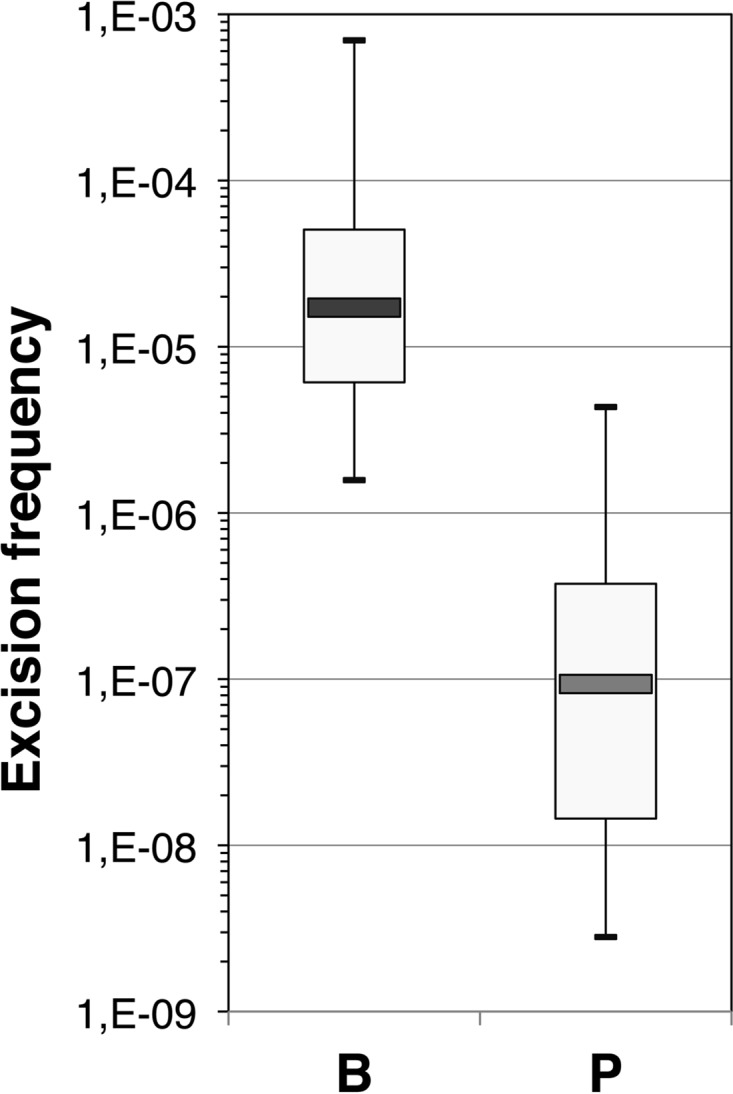
IntI1 excision activity under biofilm conditions. IntI1 excision recombination activity was estimated by determining the frequency of emergence of tobramycin resistance as a result of recombination between the *attC* sites of the *attC_aadA7_-cat*(T4)-*attC*_VCR_*-aac(6′)-Ib* gene cassette array carried on plasmid p6851. The *intI1* gene was expressed from its own promoter (pZE1-intI1). Excision frequency was estimated under biofilm conditions (B) and in planktonic culture (P) with the wild-type strain MG1656 F′ carrying both p6851 and pZE1-intI1. Assays were done at least 9 times each. The bottom and top of the box indicate the first and third quartile respectively. The median is shown as a horizontal line inside the box, and the maximum and minimum values as the ends of the whisker.

To examine the consequences of higher basal class 1 integrase expression on cassette rearrangement under biofilm conditions, we estimated the excision activity of the integrase by measuring its capacity to catalyze recombination between two *attC* sites located on a synthetic array of two cassettes: *attC_aadA7_*-*cat*(T4)-*attC*_VCR_-*aac(6′)-Ib* (12). When the *intI1* gene was expressed from the wild-type promoter PintI1 (pZE1-*intI1*) (Table 1), the cassette excision frequency rose by more than 2 log under biofilm conditions compared to the frequency in planktonic culture (average values, 1.3 × 10^−05^ versus 6.6 × 10^−07^; *P* < 0.001) (Fig. 1).

### Existence of biofilm-specific PintI1 regulation.

In the Δ*lexA* strain, the expression of the PsfiA promoter was maximal and independent of the growth conditions (biofilm or planktonic culture), as expected for a derepressed background (Table 2). Contrary to the results for PsfiA, in the Δ*lexA* strain, PintI1 exhibited a significantly higher expression level under biofilm conditions than in planktonic culture (1.5-fold difference) (Table 2), suggesting either biofilm-specific regulation of PintI1 or pleiotropic effects due to *lexA* deletion differentially affecting the PintI1 and PsfiA promoters. To test these two possibilities, we used the plasmid pPintI1* bearing the transcriptional fusion PintI1*-*lacZ*, in which the LexA box within PintI1 is mutated, inhibiting LexA binding and therefore leading to constitutive expression of *lacZ* (Table 1) (12). This construct should therefore exhibit maximum *lacZ* expression independently of both growth condition (biofilm versus planktonic) and bacterial background (WT versus Δ*lexA*). We found that the strength of PintI1* was still higher under biofilm conditions than in planktonic culture, whatever the genetic background (MG1656 F′ or its Δ*lexA* derivative) (Table 2), indicating that the difference was not due to a pleiotropic effect of *lexA* deletion.

Together, these results suggest that the expression of the class 1 integron integrase is also subjected to biofilm-specific regulation independently of the SOS response.

### Role of RelA and Lon in the regulation of *intI1* expression under biofilm conditions.

Biofilms being heterogeneous environments in which various stresses are encountered, we constructed various global regulator deletion mutants of the MG1656 F′ strain, namely, *rpoS* (general stress response), *cpxR* (envelope stress response), *luxS* (quorum sensing), *relA/spoT* (stringent response), and *lon* (protease) mutants, in order to examine their possible involvement in the regulation of *intI1* expression under biofilm conditions. We estimated the strength of the *intI1* promoter in these deletion mutants of MG1656 F′ grown under planktonic and biofilm conditions. To circumvent interference with SOS regulation, we used the PintI1* promoter, which carries a mutated LexA box (Table 1).

We first tested the ability of these mutants to form a biofilm. Apart from the *cpxR* mutant, which, compared to the parental strain, exhibited a slightly lower capacity to form a biofilm, none of the mutants showed an altered biofilm-forming capacity (see Fig. S1 in the supplemental material). We therefore evaluated PintI1* activity in all of these mutants. The biofilm/planktonic condition ratio of β-galactosidase activities in the *luxS*, *cpxR*, and *rpoS* MG1656 derivatives was similar to that of the parental strain MG1656 F′. However, in the backgrounds with deletions of *relA/spoT* and *lon*, there was no longer a significant difference between the β-galactosidase activities in biofilm and planktonic culture (Table 3).

**TABLE 3:** Activity of PintI1* under biofilm and planktonic conditions for the deletion mutants

Strain	β-Gal activity (Miller units) [mean (±SD)^a^ or B/P ratio] under indicated condition(s) of strain bearing pPintI1*-*lacZ*		
	P	B	B/P
MG1656 F′	97.6 (±23.2)	202.9 (±29.3)	1.8#
MG1656 Δ*luxS* F′	65.0 (±20.1)	138.0 (±38.5)	2.1†
MG1656 Δ*cpxR* F′	46.0 (±4.7)	81.3 (±6.2)	1.8†
MG1656 Δ*rpoS* F′	89.3 (±28.6)	182.6 (±38.0)	2.0†
MG1656 Δ*relA/spoT* F′	65.5 (±8.2)	88.0 (±17.6)	1.3, NS
MG1656 Δ*lon* F′	70.0 (±15.9)	92.8 (±22.4)	1.3, NS

a Results shown are from at least 6 replicates. P, planktonic culture; B, biofilm; # and †, significant difference at a *P* value of <0.001 or <0.01, respectively; NS, not significant.

**FIG 2:**
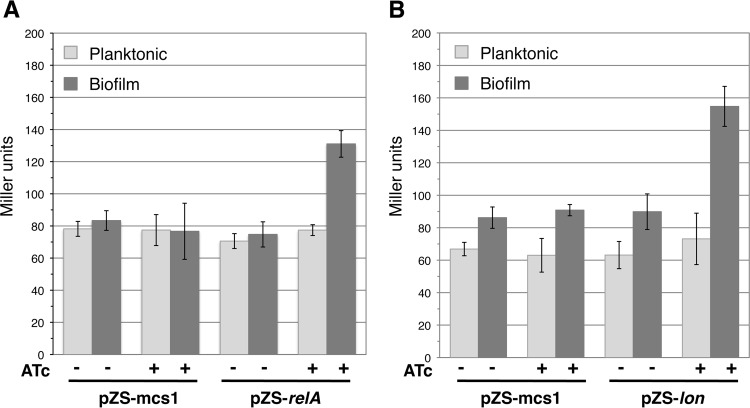
Complementation experiment with *relA* and *lon* mutants. The activity level of the derepressed integrase promoter PintI1* was estimated by β-galactosidase assay. (A) Results for complementation of *relA*. MG1656Δ*relA/spoT* F′/pPintI1*-*lacZ* also carried pZS*tetR11-mcs1 (pZS-mcs1) or pZS*tetR11-*relA* (pZS-relA). (B) Results for complementation of *lon*. MG1656Δ*lon* F′/pPintI1*-*lacZ* also carried pZS*tetR11-mcs1 or pZS*tetR11-*lon* (pZS-lon); Strains were grown for 24 h under biofilm conditions or planktonic culture in the absence or presence of 0.2 mM anhydrotetracycline (ATc; induction of RelA or Lon protein synthesis). Error bars indicate the standard deviations of the results from 6 different assays.

To confirm the role of the stringent response and of the Lon protease in the biofilm-specific regulation of *intI1*, we complemented the MG1656Δ*relA/spoT* F′ and MG1656Δ*lon* F′ mutants with RelA and Lon, respectively (Table 1) (39). As shown by the results in Fig. 2, the induction of RelA and Lon protein production, respectively, in the *relA/spoT* and *lon* deletion mutants restored the wild-type phenotype in both mutants, i.e., higher *lacZ* expression from PintI1* under biofilm conditions than in planktonic culture (1.7- and 2.1-fold differences, respectively). These results showed that PintI1* induction under biofilm conditions resulted from a direct and/or indirect effect of the stringent response and that Lon protein also played a role in this regulation.

**FIG 3:**
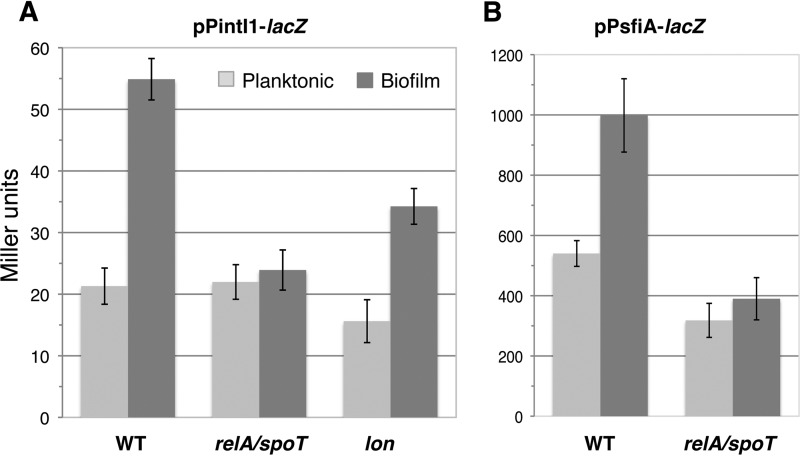
Effect of the stringent response on *intI1* and *sfiA* expression under biofilm conditions. The activity levels of the PintI1 and PsfiA promoters, expressed as Miller units, were estimated by β-galactosidase assay in 24-h planktonic and biofilm cultures of the wild-type (WT) strain MG1656 F′ and its Δ*relA/spoT* and Δ*lon* derivatives, as indicated. The bacteria carried plasmid pPintI1-*lacZ* (A) or PsfiA-*lacZ* (B)*.* Error bars indicate the standard deviations of the results from at least 6 different assays.

We then examined whether the biofilm-specific regulation observed with PintI1* also affected the wild-type SOS-regulated PintI1 promoter by estimating the β-galactosidase activities from PintI1-*lacZ* in strains MG1656Δ*relA/spoT* F′ and MG1656Δ*lon* F′. As observed with PintI1*, there was no longer any difference between the β-galactosidase activities under planktonic and biofilm conditions with the MG1656Δ*relA/spoT* PintI1-*lacZ* strain (Fig. 3). This result was surprising, as we knew from the above-described PsfiA experiments that the increase of PsfiA basal activity in biofilm compared to that in planktonic culture was SOS dependent. Thus, we expected that the increased activity of PintI1 in biofilm should also be at least partially dependent on the SOS response (Table 2). We therefore examined whether RelA was also responsible for the higher basal expression level of *sfiA* under biofilm conditions than in planktonic culture. As observed with PintI1, the β-galactosidase activity from PsfiA-*lacZ* in strain MG1656Δ*relA/spoT* F′ was similar under biofilm and planktonic conditions (Fig. 3). These results thus suggested that the stringent response might somehow induce the SOS response, which would in turn increase basal *intI1* and *sfiA* expression under biofilm conditions. In the MG1656Δ*lon* F′ background, contrary to what was observed with PintI1*, the β-galactosidase activity from PintI1-*lacZ* was 2.2-fold higher (*P* < 0.01) under biofilm conditions than in planktonic culture (Fig. 3).

## DISCUSSION

The aim of this study was to assess the expression/activity of the class 1 integron integrase IntI1 under biofilm conditions. In agreement with Bernier et al., who showed that the SOS response is gradually induced in aging static biofilm culture in minimal medium (up to twofold after 96 h) (28), we found that both the SOS response and class 1 integron integrase expression were induced more than twofold (up to 3.6-fold for *intI1*) in 24-h continuous biofilm culture in LB medium compared to their expression level in planktonic culture. We also found that the expression of *sfiA* and *intI1* was enhanced under biofilm conditions in the *lexA* deletion mutant background compared to their levels in the parental strain (up to 12.3-fold for *sfiA*). This indicates that, although the SOS response is a signature of the biofilm lifestyle, its level of induction under biofilm conditions varies with the growth conditions and does not reach its fully derepressed level, providing bacteria with some leeway to cope with exogenous stresses.

We also observed that, under derepressed conditions (*lexA* deletion mutant background or with PintI1*), the *intI1* expression level was still higher under biofilm conditions than in planktonic culture. This suggests the existence of unexpected biofilm-specific regulation of *intI1* expression, indicating that the regulation of integron integrase is more complex than previously thought. It was recently shown that, besides its regulation via the SOS response, the *V. cholerae* integron integrase IntIA is also subject to positive CRP-dependent regulation, likely fully independent of SOS regulation (19). CRP, the c-AMP receptor protein, has been implicated in the regulation not only of the catabolic pathway but also of genes involved in adaptation and survival in the environment and virulence (40, 41). Using the virtual footprint tool PRODORIC (http://www.prodoric.de), we found no CRP binding site within the In40 class 1 integron *attI* site that encompasses the *intI1* promoter (42), suggesting that PintI1 is not regulated by CRP.

In bacteria, various nucleotides [c-di-GMP, c-di-AMP, cGMP, cAMP, (p)ppGpp, etc.] have emerged as important second messengers in the regulation of key processes required for adaptation and biofilm formation (43, 44). The *E. coli* stringent response, mediated by the alarmone (p)ppGpp, is responsible for reorganizing cellular transcription in response to nutritional starvation and other stresses, ultimately reducing the growth rate (45, 46). The concentration of (p)ppGpp [denoting both ppGpp and (p)ppGpp] is governed by the two synthases RelA and SpoT, the latter protein also acting as a hydrolase. Surprisingly, the deletion of *relA* and *spoT* abrogated the induction of both PintI1 and PsfiA under biofilm conditions (Table 3; Fig. 3). (p)ppGpp regulates replication, transcription and translation (47). It induces pausing of transcription elongation at some positions, which can hamper replication (48, 49) and lead to R loop formation (reviewed in reference 50). R loop formation has been shown to induce the SOS response (51). Furthermore, transcription profiling showed that the stringent response in *E. coli* induces the SOS response (52). It is thus conceivable that the stringent response is activated under biofilm conditions, leading to mild induction of the SOS response and, thus, to the observed increases in *intI1* and *sfiA* expression compared to their expression in planktonic culture.

Our results showed that the stringent response also regulates *intI1* expression in biofilms independently of the SOS response (Table 3). RelA is a global regulator of the stringent response and cannot act directly on PintI1. (p)ppGpp also plays a role in regulating the acid stress response, facilitates the use of alternative sigma factors (such as σ^S^, σ^E^, and σ^N^), and stabilizes σ^S^, the sigma factor that is encoded by *rpoS* and controls the general stress response (for recent reviews, see references 53 and 54). The MG1656Δ*rpoS* F′ mutant exhibited higher expression from PintI1* under biofilm conditions than in planktonic culture (Table 3), indicating that σ^S^ is not the missing link between RelA/(p)ppGpp and biofilm-specific *intI1* regulation.

The stringent response also represses the activity of exopolyphosphatase (PPX), resulting in the accumulation of polyphosphate (poly-P), which binds to Lon, stimulating its protease activity toward proteins such as free ribosomal proteins and antitoxins (55, 56). Poly-P also reduces Lon activity *in vitro* (57, 58). Interestingly, *lon* deletion had an effect similar to that of *relA/spoT* deletion on PintI1* activity under biofilm conditions, i.e., no induction compared to that in planktonic culture (Table 3). Our results thus suggest that, in biofilms, by activating the stringent response through RelA, the poly-P–Lon complex would control the amount of a biofilm-specific PintI1 regulator. Things may not be so simple, however, as *lon* deletion had no effect on the PintI1 expression level under our biofilm conditions (Fig. 3), suggesting that Lon-mediated regulation is not active when LexA is bound to PintI1. This implies that an unknown regulator of PintI1, the stability of which would be controlled by the poly-P–Lon complex, might display steric interference with bound LexA.

As biofilms are heterogeneous environments, only bacteria within certain microniches might experience nutrient starvation (59) and therefore be subject to (p)ppGpp regulation. In this case, PintI1 expression in a fraction of the biofilm population might be even higher than the global level found here. (p)ppGpp has been shown to be important for the formation of *E. coli* and *Pseudomonas aeruginosa* persisters in both planktonic and biofilm culture (60–63). Maisonneuve et al. demonstrated that the degradation of antitoxins by the poly-P–Lon complex in type II toxin-antitoxin (TA) modules is pivotal to *E. coli* persistence (62). These and our results raise the possibility that biofilm-specific induction of integrase expression might take place in persister cells.

**FIG 4:**
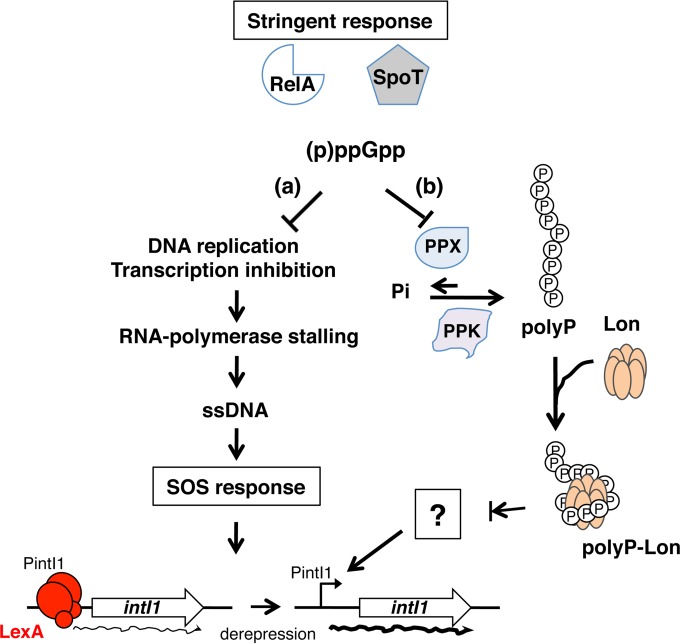
Proposed mechanism of class 1 integron regulation via stringent response under biofilm conditions. In biofilms, upon nutrient starvation, the alarmone (p)ppGpp, synthesized by the RelA and SpoT proteins, would mediate the inhibition of replication initiation and transcription of specific genes, thereby stalling the RNA polymerase and leading to the generation of single-stranded DNA (ssDNA) and, thus, to mild induction of the SOS response, resulting in autoproteolysis of the LexA dimer bound to the PintI1 promoter and expression of *intI1* (a) and the inhibition of the exopolyphosphatase (PPX) activity, resulting in the accumulation of inorganic polyphosphate (poly-P) via the polyphosphate kinase (PPK), whereupon poly-P would bind the Lon protease to form the poly-P–Lon complex that would regulate the degradation of an unknown regulator of derepressed PintI1 (b).

This study demonstrates that the regulation of class 1 integron integrase expression is more complex than previously thought, as summarized in Fig. 4. In the ubiquitous and natural settings represented by biofilms, some bacteria experience nutrient starvation that triggers a stringent response. The resulting increase in the (p)ppGpp concentration induces (i) a moderate increase in the SOS response, leading to increased basal expression of LexA-regulated genes, and (ii) biofilm-specific positive regulation of class 1 integron integrase expression through the poly-P–Lon complex.

This work confirms that biofilms are environments favorable to integron-mediated acquisition/exchange of antibiotic resistance determinants through specific regulation of class 1 integron integrase. Moreover, metagenomics studies have shown that class 1 integrons may also be found on the chromosomes of environmental bacteria (64). These class 1 integrons contain a huge diversity of gene cassettes, mostly of unknown function, potentially providing adaptive functions to bacteria (37, 65). Besides antibiotic resistance, our study thus indicates that biofilms are ideal niches for shaping bacterial evolution through the exchange of gene cassettes.

## MATERIALS AND METHODS

### Strains and growth conditions.

The bacterial strains and plasmids used in this study are listed in Table 1. The F′^Tet^ factor, designated F′ for convenience, was introduced into *E. coli* MG1656 and its derivatives by conjugation, using the commercial strain NEB 5-alpha F′ as the donor.

Cells were grown under planktonic or biofilm conditions at 37°C in Luria-Bertani (LB) medium supplemented when necessary with kanamycin (Km; 25 µg ⋅ ml^−1^), ampicillin (Amp; 100 µg ⋅ ml^−1^), tetracycline (Tet; 7.5 µg ⋅ ml^−1^), zeocyn (Zeo; 30 µg ⋅ ml^−1^), or chloramphenicol (Cm; 25 µg ⋅ ml^−1^).

### Biofilm and planktonic culture.

Biofilms were produced by culturing bacteria at 37°C in LB medium for 24 h in a continuous-flow glass microfermentor containing a removable spatula, as described in reference 38. The microfermentors were inoculated by dipping the removable glass slides for 2 min into 15 ml of bacterial culture containing 1× 10^9^ cells/ml, followed by a brief rinse in LB medium before insertion in the microfermentor. After 24 h of growth under nonbubbling conditions, the biofilm that formed on the removable glass slide was resuspended in 10 ml of ice-cold LB by vortexing. Biofilm biomass was estimated by determining the optical density at 600 nm (OD_600_).

For planktonic culture, 100 µl of the culture used to inoculate the microfermentors was diluted in 10 ml of LB and grown for 24 h at 37°C with shaking.

### Mutant construction.

MG1656Δ*gene*::KmFRT strains [“gene” denotes *relA*, *cpxR*, *rpoS*, *luxS*, or *lon*, and KmFRT is the resistance cassette used to replace a gene of interest, composed of the *aph(3′)-II* gene (Km resistance) flanked on each side by a FRT site (specific recombination site of the FLP recombinase of *Saccharomyces cerevisiae*)] were created by P1*vir* transduction from strain TG1Δ*gene*::KmFRT into MG1656 F*′*. The Km resistance gene was then removed by flippase action (66) to obtain strain MG1656Δ*gene* F′.

Constructs were verified by PCR and sequencing (Applied Biosystems 3130XL Genetic Analyser). All primers are listed in Table S1 in the supplemental material.

### Plasmid construction.

pPsfiA-*lacZ* was constructed by amplifying the *sfiA* promoter, PsfiA, from MG1656 genomic DNA with PCR using primers psulA-3 and psulA-EcoRI-5 and cloning the product into pSU38Δtot*lacZ* at the EcoRI/BamHI sites.

pZS*tetR11-*relA* was constructed as follows: *relA* was amplified from the MG1656 genome by using primers relA-KpnI-5 and relA-HindIII-3 and cloned via KpnI/HindIII into pZS*21mcs1 (39), yielding pZS*21-*relA*. The *neo* gene (kanamycin resistance) from pZS*21-*relA* was replaced by the *bla* gene of pZE1-mcs1 by XhoI/SacI cloning, yielding pZS*11-*relA*. *tetR* was amplified with its P_N25_ promoter from pZEtetR21-gfp (67) using primers tetR-SacIinfu-3 and tetR-SacIIinfu-5 and cloned at the SacI site of pZS*11-*relA* by using the In-Fusion method (In-Fusion HD cloning kit, Clontech), following the manufacturer’s instructions, to yield pZS*tetR11-*relA*.

pZS*tetR11-*lon* was constructed by using the in-Fusion approach to replace *relA* with *lon.* The *lon* fragment was amplified from MG1656 by using lon-infusion-3′ and lon-infusion-5′ primers and cloned with linearized pZS*tetR11-*relA* (KpnI/HindIII), following the manufacturer’s instructions.

pZS*tetR11-mcs1 was constructed as follows: the XhoI/SacI fragment from pZS*tetR11-*relA* containing *tetR* was cloned into pZS*21-mcs1, replacing the *neo* gene with the *tetR-bla* fragment.

All constructs were verified by sequencing. All primers are listed in Table S1 in the supplemental material.

### β-Galactosidase assay.

The β-galactosidase assay was performed with 0.5-ml aliquots of planktonic culture or 0.5 ml of resuspended biofilm, as described in reference 68.

### Cassette excision assay.

A synthetic array of two cassettes [*attC_aadA7_*-*cat*(T4)-*attC*_VCR_*-aac(6′)-Ib*], preceded by the *lac* promoter, P*lac*, and conferring chloramphenicol resistance [*cat*(T4)], is carried on plasmid p6851. The excision assay is described in reference 12. Briefly, MG1656 F′/p6851 cells electroporated with pZE1-IntI1 or pZE1-IntI1* (Table 1) were grown overnight in LB medium. These cultures were used to inoculate both planktonic and biofilm cultures, which were then grown for 24 h. Dilutions of resuspended biofilm or planktonic culture were plated on LB-Amp-Km plates (total population) and LB-tobramycin (Tobra) plates (recombinants only). The excision frequency was calculated by determining the ratio of Tobra^r^ to Amp^r^ Km^r^ colonies (CFU/ml). Experiments were performed at least 9 times.

### Statistical analysis.

Significance was determined using the nonparametric Mann-Whitney *U* test to compare the results under the two experimental conditions (biofilm and planktonic) and for the wild-type and mutant strains. *P* values of <0.05 were considered to indicate statistical significance.

## SUPPLEMENTAL MATERIAL

Figure S1  Biofilm formation by mutants. Download Figure S1, PDF file, 0.1 MB

Table S1  List of primers and probes used in this study. Table S1, PDF file, 0.05 MB
