# Supplementary material for: The Stringent Response Promotes Antibiotic Resistance Dissemination by Regulating Integron Integrase Expression in Biofilms
Source: mBio. 2016 Aug 16;7(4):e00868-16. doi: 10.1128/mBio.00868-16 (PMC4992968; doi:10.1128/mBio.00868-16)
Supplement: Figure S1 — Biofilm formation by mutants. Download [file mbo004162936sf1.pdf]

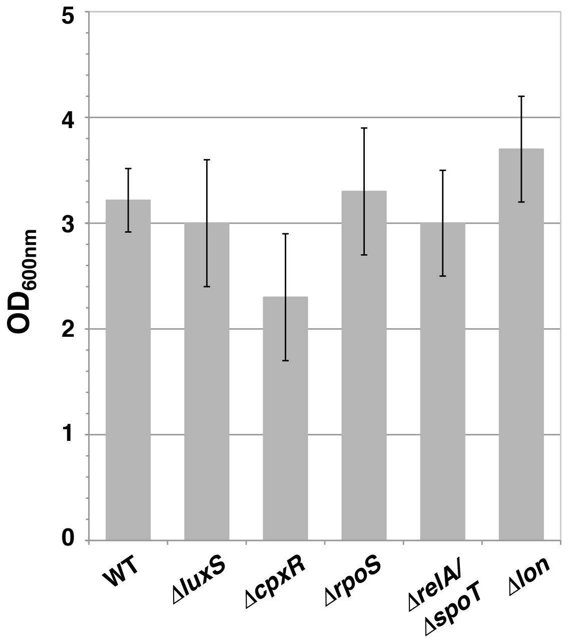

Fig. S1: Biofilm formation by mutants

Biofilms of WT strains MG1656 F' and its derivatives were grown for 24-h and resuspended before reading OD_{600nm}. Error bars indicate the standard deviation for at least 6 different assays.
