# Supplementary material for: The Stringent Response Promotes Antibiotic Resistance Dissemination by Regulating Integron Integrase Expression in Biofilms
Source: mBio. 2016 Aug 16;7(4):e00868-16. doi: 10.1128/mBio.00868-16 (PMC4992968; doi:10.1128/mBio.00868-16)
Supplement: Table S1 — List of primers and probes used in this study. [file mbo004162936st1.pdf]

Table S1. List of primers and probes used in this study

Name	sequence (5'>3')
psulA-3	GAAGGATCCATAATCAATCCAGCCCC
psulA-EcoRI-5	ACGGAATTCTTAACTACGAAAATAGGC
rpoS-ext3	AGCCGCATTTATTATTTC
rpoS-ext5	GCAAGCACAACCGAGCCGAC
luxS-ext3	TTCTTTATGCCGCTGACC
luxS-ext5	GAAACGCGTCGCGCAAACG
cpxR-ext3	ACTGCTGGCCGGACGAATC
cpxR-ext5	GCCATCTCAACCTGACGAG
lon-ext3	CTGCACGGAAGCTCGGTAC
lon-ext5	CAGATCCTCAAAGAGCCGA
relA-ext3	ATCAACCCAAATCAGATC
relA-ext5	GCTGGTGGAAAAAGGCCAG
spoT-ext3	AGGCAGGCCTTGCGTTTTGCG
spoT-ext5	CGCGGAGTATCTTTATTTTACC
Km-verif-5	CAGTCATAGCCGAATAGCCT
Km-verif-3	GGATTCATCGACTGTGGCCG
tetR-SacIIinfu-5	TCAGACCAAGTTTACGCCGCGGGCGCAACGCAATTAATGTAAGTTAG
tetR-SacIinfu-3	AACAGGAGTCCAAGCGAGCTCGCTTAAGACCCACTTTCACA
relA-HindIII-3	TTTAAGCTTAACTCCCGTGCAACCGAC
relA-KpnI-5	GAGGGTACCATGGTTGCGGTAAGAAGTG
relA-rev2	CTGGAACATCTGGGGATCA
relA-for2	ATCAGGTTGCCAACACCTTC
lon-infusion-5’	ATTAAAGAGGAGAAAGGTACCGCATGAATCCTGAGCGTTCTG
lon-infusion-3'	CAGGAATTCGATATCAAGCCTATTTTGCAGTCACAACCT
lon-for2	GCATTATTCGTTACTAC
lon-rev-2	CGAATAATGCCGATAATG
ApXFP-5	ATTCTCACCAATAAAAAACGC
ApXFP-3	GCGGCGGATTTGTCCTACTCA
TetR-verif-5	TAAAGTAAAATGCCCCAC
TetR-verif-3	TAGAAGGGGAAAGCTGGCA
pZS*-TetR-rev	GTGATAGAGATACTGAGC
